# Validation of the ELAN-HF Score and self-care behaviour on the nurse-led heart failure clinic after admission for heart failure

**DOI:** 10.1186/s12912-022-00914-1

**Published:** 2022-06-21

**Authors:** T. A. M. Vinck, R. Deneer, CCAG Verstappen, WE Kok, K. Salah, V. Scharnhorst, LC Otterspoor, The Severity of bioMArkers and Self-care in Heart failure

**Affiliations:** 1 Department of Cardiology Catharina Hospital, Eindhoven, the Netherlands; 2 Clinical Laboratory, Catharina Hospital, Eindhoven, the Netherlands; 3 Eindhoven University of Technology, Eindhoven, the Netherlands; 4 Expert Center Clinical Chemistry Eindhoven, Eindhoven, the Netherlands; 5 Amsterdam UMC, University of Amsterdam, Heart Center; department of Clinical and Experimental Cardiology, Amsterdam, the Netherlands; 6 Department of Radiology and Nuclear Medicine, Radboud University Medical Centre Nijmegen, Nijmegen, the Netherlands

**Keywords:** Nurse-led heart failure clinic, Nurse practitioner, Specialized nurse, Nursing, Heart failure, NT-proBNP, Self-care behaviour, Risk of readmission, Discharge, ELAN-HF score

## Abstract

**Aim:**

To validate the predictive value of the European coLlaboration on Acute decompeNsated Heart Failure (ELAN-HF) score, and to assess the effect of self-care behaviour on readmission and mortality in patients after admission with acute decompensated heart failure (ADHF).

**Design:**

Quantitative, prospective, single centre, cohort study.

**Methods:**

N-Terminal pro–B-type natriuretic peptide (NT-proBNP) levels were measured on admission and discharge, and were used together with clinical and laboratory parameters to calculate the ELAN-HF score. Patients were stratified into four risk groups (low, intermediate, high, very high) according to their ELAN-HF score. The performance of the ELAN-HF score was evaluated and compared to the original study. Self-care behaviour was assessed by the European Heart Failure Self-care Behaviour Scale (EHFScBS-9). Survival analysis was used to estimate the association between both scores and re-admission for HF and/or all-cause mortality within 180 days.

**Results:**

88 patients were included. The median age of the study population was 75 years (IQR 69–83), 43% was female. NYHA III/IV functional class was present at discharge in 68 patients (85%) and 27 patients (34%) had a left ventricular ejection fraction < 40%. Complete data and 180 day follow up was available for 80 patients. 55% reached the endpoint of readmission and/or all-cause mortality. There was a significant association between the ELAN-HF score and re-admission and/or mortality < 180 days (HR = 1.25, 95% CI 1.08—1.45, *p* = 0.003). The median EHFScBS-9 score was 68.1 (IQR 58.3 – 77.8). There was no significant association between the EHFScBS-9 score and readmission and/or mortality < 180 days (HR = 1.01, 95% CI 0.99—1.03, *p* = 0.174).

**Conclusion:**

This study confirms the validity and therefore the potential of the ELAN-HF score to triage patients with ADHF before discharge. Using this score may optimize the follow-up treatment on the nurse-led heart failure clinic in order to decrease readmission and mortality. Self-care behaviour was non-significantly associated with readmission and/or mortality in our study population.

**Trial Registration:**

This study has been registered with the ethics committee MEC-U (Nieuwegein, The Netherlands), registration nr: V.160999/W18.208/HG/mk.

## Introduction

Heart failure (HF) is defined by the *European Society of Cardiology* as a clinical syndrome and an inadequacy of the pumping function of the heart, characterized by symptoms such as shortness of breath, persistent coughing or wheezing, ankle edema and fatigue, that may be accompanied by the following signs: elevated jugular venous pressure, pulmonary crackles, increased heart rate and peripheral oedema [1].

HF is a major health problem, with a prevalence of 230.200 patients per year and a mortality rate of 7.689 patients per year in the Netherlands [2].

At present, approximately 26 million people worldwide are living with HF. The prognosis of patients suffering from HF is poor, with survival rates worse than those for bowel, breast or prostate cancer [3]. HF causes severe economic, social and personal costs. Globally, the increasing burden of HF is taking its toll on society, in particular on patients, caregivers and healthcare systems [3].

After hospitalization for acute decompensation, approximately 20% of patients with HF are readmitted within 30 days and over 50% within 6 months with a 60-day mortality rate after admission is 15.2% [4].

Nurses on the nurse-led HF clinic play a crucial role in the prevention of readmission and mortality in patients with HF. In order to improve this care, accurate prediction scores on these events may be of great value.

A common measure in the nurse-based HF clinic, in order to reduce readmission and mortality, is patient education in self-care behaviour [5]. The present study addresses both the prediction of readmission and mortality and self-care behaviour after an admission for HF.

## Background

### Nurse-led HF outpatient clinics

The concept of HF nurses working in an outpatient clinic was for first time described in 1983 [6]. This was followed by the first nurse‐led HF clinic started in Sweden in 1990 after which they spread out to many Swedish hospitals. Nurse‐led HF clinics reduce the need for hospital care since titration of drugs can be rapidly achieved. Furthermore, studies indicate that early follow‐up after hospitalization may prevent readmissions [6].

Similar to these successes on HF clinics, several studies on other nurse-led clinics also indicated positive effects [7]. Rich et al. investigated the effect of a multidisciplinary, nurse‐directed intervention and found that the intervention improved the patients’ compliance, quality of life and decreased the rate of readmission and the healthcare costs [8]. Nurses independently perform anamnesis and physical examination, and are responsible for the diagnostic processes.

Nurse-led HF clinics provide education on self-care and psychosocial support to patients and their family. Programs employing multidisciplinary teams and in-person communication led to fewer HF hospital readmissions [9]. High-risk HF patients (advanced stage, low self-care skills, elderly, and those with frequent readmissions) may be expected to benefit the most from improvements in HF self-care knowledge and home care behaviour skills [9].

### Risk of readmission; ELAN HF score

Prognostication of patients is useful in triaging patients during and after hospitalization [10, 11]. For this purpose, specific predictors for readmission of patients with HF have emerged [12]. By combining different clinical and laboratory parameters in a clinical prediction model, patients can be triaged just before discharge. In patients with HF plasma biomarkers *Brain Natriuretic Peptide* (BNP) and *N-terminal pro-B-type Natriuretic Peptide* (NT-proBNP) are commonly used. They indicate the severity of congestion and cardiac dysfunction and predict morbidity and mortality [13].

Various risk models for readmissions and mortality in HF have already been developed [11, 14–17]. They incorporated the natriuretic peptide levels, measured either at admission or discharge, while some models also use their change during hospitalization. The PRIMA II trial, that investigated the influence of changing of NT-proBNP with guided therapy and intensified HF care pre-discharge, did not demonstrate to improve prognosis in these patients [18]. This study may have been hampered by a relatively overestimated expected benefit in the third of patients who were guided after having not reached the expected drop in NT-proBNP levels [18, 19].

The ELAN-HF (European collaboration on Acute decompensated Heart Failure) score is a model which is different from other risk models because it incorporates absolute discharge NT-proBNP levels, but also the percentage change in NT-proBNP, along with clinical risk markers [14]. Although it has been already validated retrospectively in a cohort of 325 patients, multiple external validations are needed to generalize the ELAN-HF score as a prediction model before it can be implemented in the clinical practice of the nurse-led HF clinic [20, 21].

### Self-care behaviour

Self-care behaviour is defined as the behaviour that consists of the decisions and strategies that a person undertakes for the sake of livelihood, healthy functioning and well-being [22, 23], as shown in Table 1. Previous studies indicated that optimal self-care behaviour can lead to fewer hospital admissions for HF [24]. Furthermore, is has been prospectively demonstrated that the use of information to improve self-care in HF led to a 30% decrease in readmission and outpatient visits within 30 days of discharge [24].

**Table 1:** the European Heart Failure Self-care Behaviour Scale (EHFScBS-9)

	Totally agree	Agree	Neutral	Disagree	Totally disagree
1. I weigh myself every day	1	2	3	4	5
2. If SOB (shortness of breath) increases I contact my doctor or nurse	1	2	3	4	5
3. If legs/feet are more swollen, I contact my doctor or nurse	1	2	3	4	5
4. If I gain weight more than 2 kg in 7 days, I contact my doctor or nurse	1	2	3	4	5
5. I limit the amount of fluids	1	2	3	4	5
6. If I experience fatigue, I contact my doctor or nurse	1	2	3	4	5
7. I eat a low-salt diet	1	2	3	4	5
8. I take my medication as prescribed	1	2	3	4	5
9. I exercise regularly	1	2	3	4	5

Self-care behaviour can be scored by using the European Heart Failure Self-care Behavioural Scale (EHFScBS-9), containing 9 items grouped around consulting behaviours and adherence with the regimen. Each of the items is graded with a 5-point Likert scale. This questionnaire was validated in several countries and was improved in 2014 [5].

## The Study

### Aims

The first aim of this study was to validate the predictive value of the ELAN-HF score on readmission and/or mortality in a prospective study of a hospitalized HF population.

The second aim was to assess the effect of self-care behaviour on readmission and mortality in these patients.

### Design

We conducted a quantitative, prospective, single centre cohort study. The primary endpoint is a composite endpoint of re-admission and /or all-cause mortality at 180 days.

The secondary endpoint is all-cause mortality at 180 days. Patients hospitalized for ADHF were included for three months (October—December 2017, Fig. 1).

**Fig. 1:**
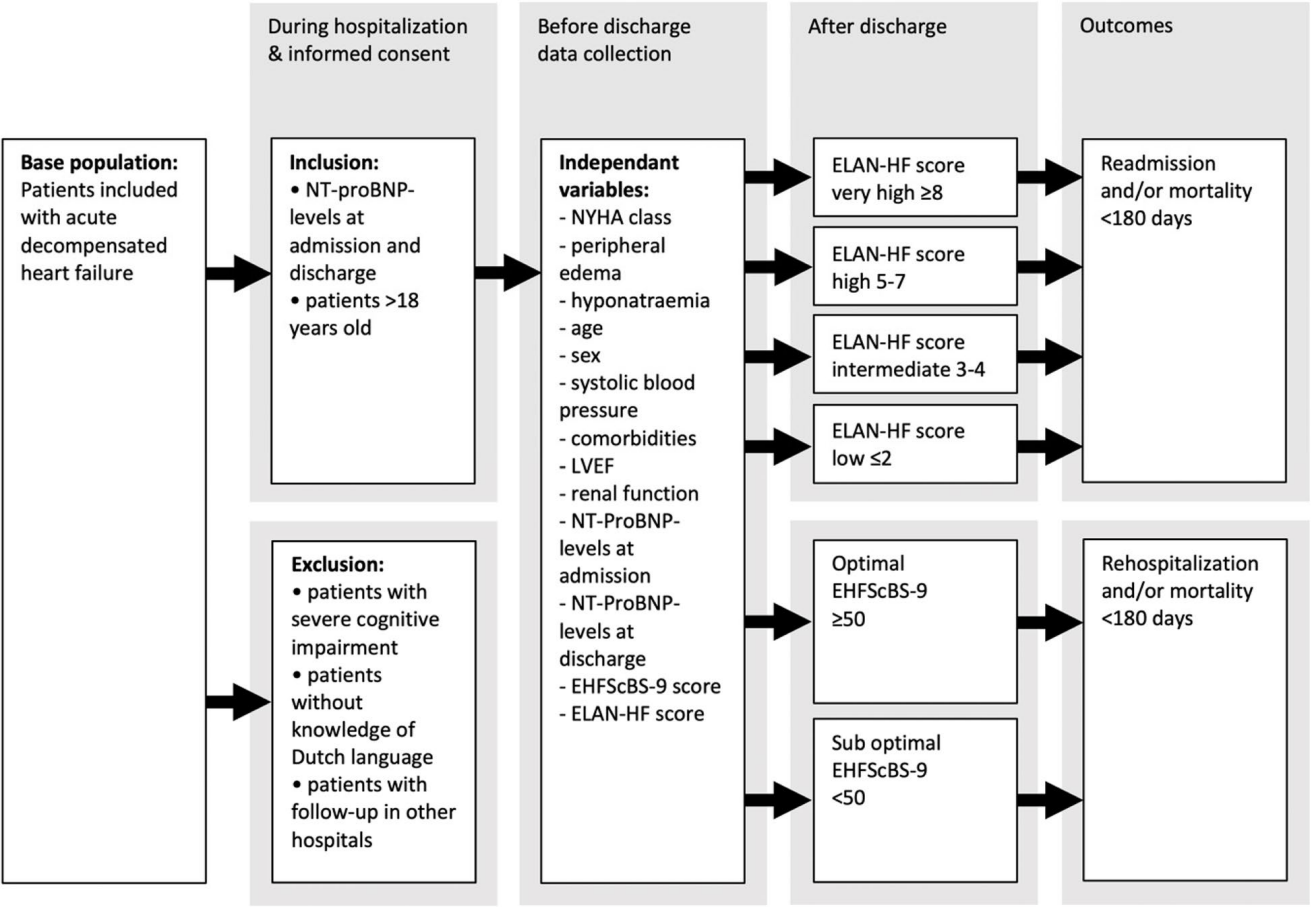
Flow chart study design. *NYHA, New York Heart Association; LVEF, left ventricular ejection fraction; NT-proBNP N-terminal pro-B-type natriuretic peptide; ELAN-HF, European collaboration on Acute decompensated Heart Failure; EHFScBS-9, European Heart Failure Self-care Behaviour Scale

A readmission was defined as an urgent clinical admission with a duration of at least 24 h after a previous discharge from the hospital.

Two sources were used for data collection. The data for both the outcome variables (readmission and / or mortality within 180 days), as well as the baseline characteristics and biomarkers (independent variables), were extracted from the electronic patient file, whereas missing data on mortality were retrieved from the general physician. The ELAN-HF score was calculated with this data (Table 2).

**Table 2:** Calculation of ELAN-HF (European collaboration on Acute decompensated Heart Failure) score

Predictor	Score	Regression coefficient
NT-proBNP reduction, % < 30 (dynamic change)	1	0.511
NT-proBNP discharge value, pg/ml		
1500–5000	1	0.713
5001–15,000	3	1.426
> 15,000	4	1.776
Age at admission, ≥ 75 years	1	0.345
Peripheral oedema at admission Yes	1	0.517
SBP at admission mmHg ≤ 115	1	0.431
Hyponatremia at admission mmol/L < 135	1	0.374
Serum Urea at discharge mmol/L ≥ 15	1	0.486
NYHA class at discharge, III/IV Yes	1	0.403
**Risk score groups ELAN-HF**		
Low (1)	≤ 2	
Intermediate (2)	3–4	
High (3)	5–7	
Very high (4)	≥ 8	

Maximum “penalty points” in the risk score is 11. Hyponatremia is equivalent to a sodium content of < 135 mmol / L; NT-proBNP, N-terminal pro B-type natriuretic peptide; SBP, systolic blood pressure; NYHA, New York Heart Association classification (extracted from the patient files)

### Participants

Patients with ADHF were included at admission. Excluded were patients with cognitive limitations, inability to speak the Dutch language and patients who were unable of follow-up.

### Ethical considerations

The local ethics committee of the Catharina Hospital approved the study. All investigators adhered to the principles of the Declaration of Helsinki. The measurements performed during this study were part of routine care. In addition, all included patients were informed orally and in writing by the investigator and gave written consent.

### Data-collection ELAN-HF score

The ELAN-HF score, as shown in Table 2, is categorized by Salah et al. into four risk categories (low, intermediate, high and very high) which corresponds to increasing 6-month mortality rates (3.6%, 9.2%, 23.5% and 51.1%).

### Data-collection self-care behaviour

To assess self-care behaviour, the Dutch version of the European Heart Failure Self-care Behavioural Scale (EHFScBS-9), was used [25]. The questionnaire was handed out by the investigator during admission and was completed by the patient without the presence of the investigator. The EHFScBS-9 score ranges from 9 – 45, but has been standardised to a scale of 0 – 100 with higher scores indicating better self-care [5]. The standardised score can be obtained by using the SPSS syntax provided by the study of Vellone et al. and also for the interpretation of which a cut-off point < 50 means sub-optimal score of self-care, a score 50–100 can be seen as an optimal/ good score of self-care [5].

### Statistical analysis

The statistical analyses consisted of two parts: (1) external validation of the ELAN-HF score in our study cohort (2) and survival analysis of the ELAN-HF score, EHFScBS-9 score and other clinically relevant variables.

### External validation

External validation is the process of evaluating model performance in a sample independent of that used to develop the model. The outcome used for external validation was 6-month all-cause mortality, analogous to Salah et al. [14]. The 6-month mortality rates for the four risk groups as reported by Salah et al. were compared to those of our study cohort. The external validation steps performed in this study are described in more detail by Royston et. al 2013 [26]. First, the ELAN-HF linear predictor was calculated by using the regression coefficients in Table 2. The ELAN-HF linear predictor was then used as a covariate in a Cox PH model. A likelihood-ratio (LR) test was performed to test whether the slope of the ELAN-HF linear predictor was equal to 1. Secondly, the model misspecification was tested formally by running a Cox PH model on all the ELAN-HF covariates and constraining the coefficient of the ELAN-HF linear predictor to 1. Thirdly, the discriminative ability of the ELAN-HF score was evaluated using Harrell’s c-index. Finally, calibration was evaluated for predicting all-cause mortality at 6-months. The baseline-hazard at 6 months was obtained through personal correspondence with the authors of the ELAN-HF paper [14]. Patients were grouped based on expected/predicted probabilities and observed probabilities were calculated. Plotting expected versus observed probabilities yielded a calibration plot.

### Survival analysis

Survival curves were analysed for the ELAN-HF and EHFScBS-9 scores and compared with log-rank tests. For the ELAN-HF the score categories described by Salah et.al were used as reference. In case of the EHFScBS-9 score, patients with an EHFScBS-9 normalized score lower than or equal to the median EHFScBS-9 normalized score were categorized as “low”, and patients above the median as “high”. Kaplan–Meier (KM) curves were analysed using the log rank test to assess if there were significant differences between groups in cumulative incidence of events. An event was defined as time to first readmission or time till death from any cause.

Secondly, survival was analysed by Cox proportional hazards (PH) models by using time to readmission or all-cause mortality within 6 months as the outcome.

Univariate Cox PH models were fit to a subset of clinically relevant variables that were not in the ELAN-HF score. The variables that were tested significant in univariate analysis were then included in a multivariate Cox PH model to assess whether the ELAN-HF score could be improved by the EHFScBS-9 score or other variables. Statistical analyses were performed in R version 4.0.3 and a p-value < 0.05 was considered statistically significant.

### Validity, reliability and rigour

The ELAN-HF score consists of clinical variables (age, peripheral oedema on admission, SBP and NYHA class) and biomarkers (NT-proBNP, sodium and urea). All variables were collected from the electronic patient file. Biomarkers were measured in the clinical laboratory on a Cobas 8000 Pro (Roche Dx, Basel, Switzerland) instrument.

The reliability and validity of the EHFScBS-9 to measure self-care has been extensively researched in multiple studies [16, 27, 28]. The studies show that the psychometric properties of the EHFScBS-9 are satisfactory.

## Results

Eighty-eight patients fulfilled inclusion of whom 8 patients were not included in the analyses due to lack of follow-up after discharge. Baseline characteristics are demonstrated in Table 3. The median age was 75 years (IQR 69–83), 38 patients (47.5%) were diagnosed with atrial fibrillation at admission and 41 patients (51.2%) had a history of ischemic heart disease.

**Table 3:** Baseline characteristics

	**ELAN-HF score low or intermediate**	**ELAN-HF score high or very high**	**Overall**
N	29	51	80
Gender = Female (%)	10 (34.5)	24 (47.1)	34 (42.5)
Age (mean (SD))	72.2 (10.7)	76.5 (8.5)	74.9 (9.5)
BMI kg/m2 (mean (SD))	26.6 (5.5)	27.1 (6.2)	26.9 (5.9)
History of DM (%)	6 (20.7)	13 (25.5)	19 (23.8)
History of COPD (%)	5 (17.2)	8 (15.7)	13 (16.2)
Atrial fibrillation at admission (%)	13 (44.8)	25 (49.0)	38 (47.5)
Admitted with ADHF in past year (%)	7 (24.1)	19 (37.3)	26 (32.5)
History of valvular disease (%)	20 (69.0)	34 (66.7)	54 (67.5)
Ischaemic aetiology (%)	14 (48.3)	27 (52.9)	41 (51.2)
Polyclinic patient (%)	1 (3.4)	15 (29.4)	16 (20.0)
NYHA class at discharge (%)			
II	5 (17.2)	7 (13.7)	12 (15.0)
III	19 (65.5)	31 (60.8)	50 (62.5)
III-IV	5 (17.2)	13 (25.5)	18 (22.5)
Left Ventricular Ejection Fraction (%)			
Preserved	11 (37.9)	20 (39.2)	31 (38.8)
Moderately reduced	11 (37.9)	11 (21.6)	22 (27.5)
Reduced	7 (24.1)	20 (39.2)	27 (33.8)
NT-proBNP at admission pg/ml (median [IQR])	3440.0 [2617.0, 5241.0]	6781.0 [3884.5, 14,211.5]	5604.0 [3038.5, 10,005.2]
NT-proBNP at discharge pg/ml (median [IQR])	1892.0 [728.0, 2376.0]	5942.0 [3056.5, 10,968.0]	3505.0 [1911.5, 7860.8]
NT-proBNP change % (mean (SD))	-57.9 (24.3)	-6.1 (54.2)	-24.8 (51.9)
ELAN-HF score (median [IQR])	3.0 [2.0, 4.0]	6.0 [5.0, 7.0]	5.0 [3.8, 6.0]
ELAN-HF score risk category (%)			
Low	10 (34.5)	0 (0.0)	10 (12.5)
Intermediate	19 (65.5)	0 (0.0)	19 (23.8)
High	0 (0.0)	41 (80.4)	41 (51.2)
Very high	0 (0.0)	10 (19.6)	10 (12.5)
EHFScBS-9 score normalized (median [IQR])	61.1 [50.0, 75.0]	69.4 [61.1, 77.8]	68.1 [58.3, 77.8]
Outcome (%)			
Event-free	20 (69.0)	16 (31.4)	36 (45.0)
Readmission	6 (20.7)	17 (33.3)	23 (28.7)
Mortality	3 (10.3)	6 (11.8)	9 (11.2)
Readmission and mortality	0 (0.0)	12 (23.5)	12 (15.0)

*BMI* based on clinical measurements weight en length, *SD* standard deviation, *COPD* Chronical Obstructive Pulmonary Disease, *DM* Diabetes Mellitus, *NYHA* New York Heart Association; *LVEF* left ventricular ejection fraction, *NT-proBNP* N-terminal proB-type natriuretic peptide in pg/ml, *IQR* Interquartile range; *ELAN-HF* European collaboration on Acute decompensated Heart Failure, *EHFScBS-9* European Heart Failure Self-care Behaviour Scale

Twenty-six patients (32.5%) had been previously hospitalized for ADHF in the penultimate year. After 180 days, more than half (*n* = 44, 55%) of the patients had an event. Thirty-five patients were readmitted and twenty-one patients died, within 180 days after discharge.

### External validation

**Fig. 2:**
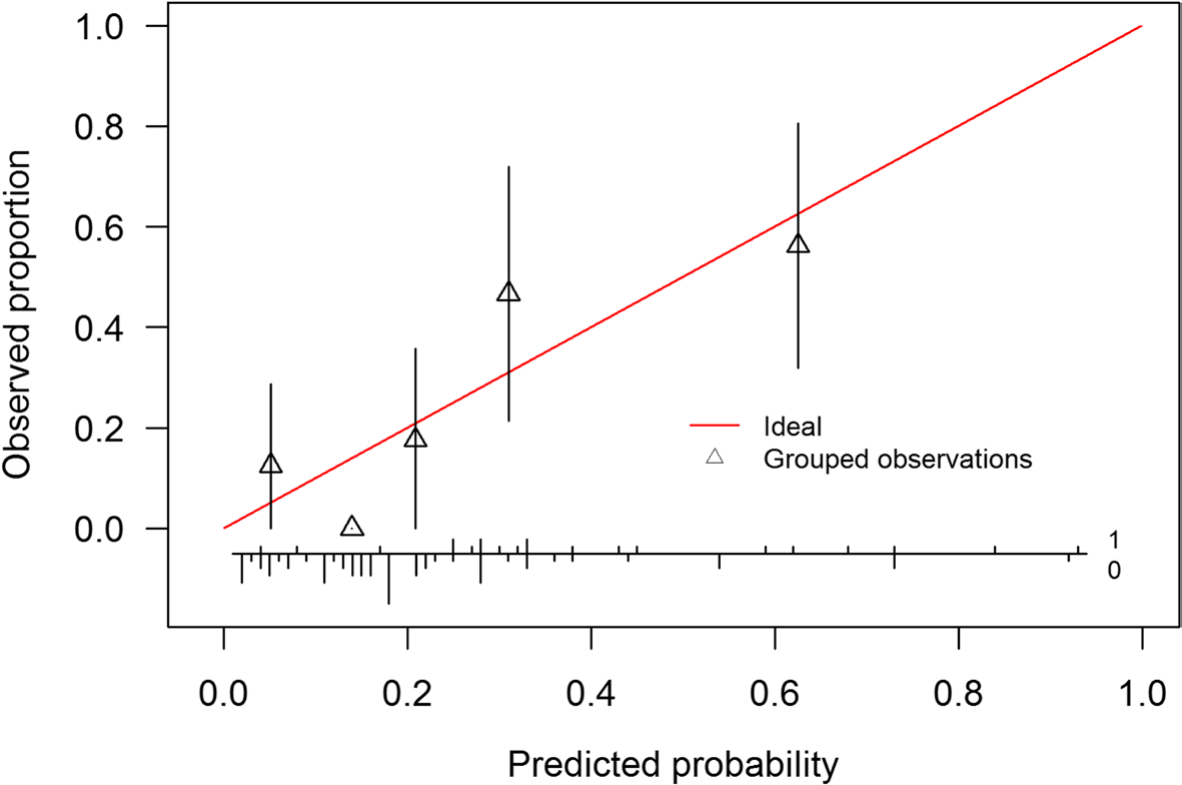
Calibration plot for predicting 6-month all-cause mortality. Observations are grouped into groups of size 16, the ideal line represents the diagonal along which there is perfect calibration. The histogram on the bottom shows the distribution of patients with (= 1) and without (= 0) an event

Table 4 presents 6-month all-cause mortality according to subdivisions of the ELAN-HF score risk groups, comparing actual and predicted mortality. Figure 2 presents the calibration plot for predicting 6-month all-cause mortality. Although some mis-calibration appears in low probabilities/risk groups, this is not statistically significant due to small number in each group. The slope of the ELAN-HF linear predictor in the validation cohort was 0.80 (SE = 0.22), the slope is not significantly different from 1 (LR test χ^2^_df = 1_ = 0.81, *p* = 0.367), so the discrimination of the ELAN-HF score seems to be preserved in our cohort. There was also no evidence of model misspecification, a joint test of all the predictors was non-significant (χ^2^_df = 10_ = 14.71, *p* = 0.143), meaning that the regression coefficients of the ELAN-HF score do not appear biased. The discriminative ability expressed in Harrell’s c-index was 0.719 (SE = 0.056) in our cohort, this is similar to the reported index by Salah et al. of 0.71 [14].

**Table 4:** 6-month mortality rates

ELAN-HF score risk group	ELAN-HF cohort	Study cohort (95% CI)	
Low ≤ 2	3.6%	10.0%	(0 – 28.8%)
Intermediate 3—4	9.2%	10.8%	(0 – 23.3%)
High 5–7	23.5%	29.3%	(13.8 – 41.9%)
Very high ≥ 8	51.1%	60.0%	(14.5 – 81.3%)

Comparison between 6-month mortality rates in the ELAN-HF development cohort and in this study cohort. If calibration is good, mortality rates should agree

### Survival analysis

**Fig. 3:**
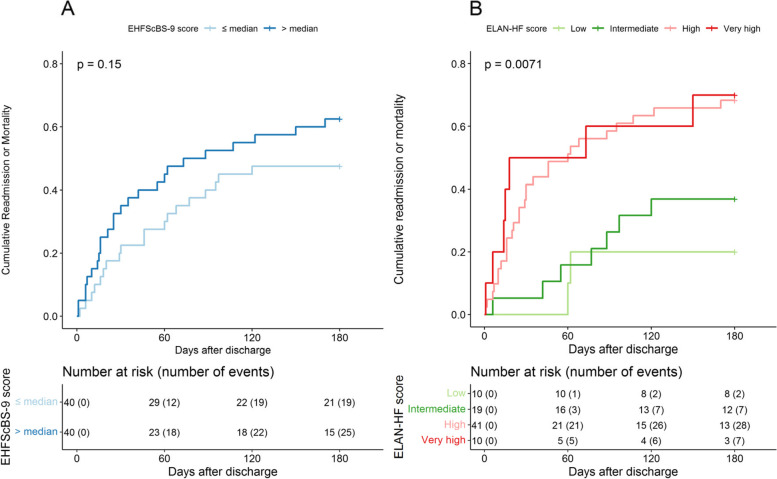
Kaplan–Meier curves. **A**: Kaplan–Meier curve for composite endpoint of readmission and/or mortality within 180 days in relation to the self-care behaviour EHFScBS-9 score. On the X-axis the time in days until the first HF readmission or all-cause mortality within 180 days. On the Y-axis the event rate in percentages. **B**: Kaplan–Meier curve for composite endpoint of readmission and/ or mortality within 180 days in relation to the ELAN-HF risk score categories. On the X-axis the time in days until the first HF readmission or all-cause mortality within 180 days. On the Y-axis the event rate in percentages

The Figs. 3A and 3B show the relationship between the risk groups derived from both EHFScBS-9 (panel A) and ELAN-HF scores (panel B) and the composite endpoint of readmission and / or all-cause mortality. There was no significant difference in composite endpoint among patients with low EHFScBS-9 score (i.e., below or equal to the median of the normalized EHFScBS-9 score) in comparison to patients with a high score (24%, versus 31% respectively KM-log rank test *p* = 0.15). Readmission and/or mortality rate was significantly higher in patients with higher ELAN-HF scores in comparison to those with low scores (KM log-rank test *p* = 0.0071). Due to the smaller sample size, there is an overlap between survival curves of the low and intermediate, and high and very high-risk groups.

Univariate Cox regression analysis for the composite endpoint results is shown in Table 5. Univariate analysis did not show that the normalized EHFScBS-9 score was associated with 6-month readmission and/or mortality. Other than the ELAN-HF score, two additional variables showed a significant association; whether the patient was admitted with ADHF in the previous year, and whether the patient is an outpatient clinic patient. Both factors increased the risk of 6-month readmission and/or mortality. This association remained significant in multivariate analysis. A LR-test revealed that adding these variables to the ELAN-HF score improved the model fit (χ^2^_df = 2_ = 10.61, *p* = 0.005) in predicting risk of 6-month readmission and/or mortality.

**Table 5:** Cox regression analysis (univariate and multivariate) for readmission and / or mortality ≤ 180 days

	**Univariate HR**	**Univariate p-value**	**Multivariate HR**	**Multivariate p-value**
Gender	1.45 (0.8 to 2.61)	0.223		
History of DM	1.05 (0.53 to 2.08)	0.888		
History of COPD	1.87 (0.92 to 3.8)	0.084		
Atrial fibrillation at admission	1.51 (0.83 to 2.73)	0.175		
Admitted with ADHF in past year	2.42 (1.33 to 4.4)	0.004	1.90 (1.02—3.54)	0.044
Outpatient clinic patient	2.78 (1.45 to 5.35)	0.002	2.16 (1.10—4.24)	0.025
Left Ventricular Ejection Fraction				
Preserved	Reference			
Moderately reduced	1.02 (0.63 to 1.66)	0.927		
Reduced	1.28 (0.73 to 2.23)	0.387		
ELAN-HF score	1.27 (1.11 to 1.46)	< 0.001	1.24 (1.085—1.44)	0.003
EHFScBS-9 score normalized	1.01 (0.99 to 1.03)	0.174		

*COPD*, Chronical Obstructive Pulmonary Disease, *LVEF*, Left Ventricular Ejection Fraction, *ELAN-HF*, European collaboration on Acute decompensated Heart Failure, *EHFScBS-9* European Heart Failure Self-care Behaviour Scale

## Discussion

### Validation of the ELAN-HF score

The ELAN-HF study retrospectively defined a risk score model for all-cause mortality for patients discharged after ADHF [14], which was validated in an independent cohort [21].

In our study, we validated the ELAN-HF score with prospectively collected data and demonstrated that 180-day mortality can be robustly predicted.

For the present analysis, we used the composite endpoint of mortality and readmission for additional analysis of the risk prediction value of the EHFScBS-9 self-care score after admissions for HF to compare with the risk prediction value of the ELAN-HF score for this endpoint. It was notable that the composite endpoint event rate within 180 days in our study was 55%, compared to 43% in the ELAN-HF study. This can be explained by the fact that patients in our study had a relatively higher NYHA-class compared to the ELAN HF study. While the ELAN-HF score was retained in the model as a predictor of the composite endpoint, the EHFScBS-9 self-care score was not.

Based on these results, we think that implementing the ELAN-HF risk model on the nurse-led HF clinic may offer more guidance to follow-up of these patients and we strongly suggest to add this score to the discharge checklist as standard care. Possible consequences may be that the high-risk population can benefit from more aggressive treatment and also from a much closer follow-up by intensive (tele-) monitoring throughout the entire HF care network.

### Self-care behaviour and prognosis

While earlier studies demonstrated a relationship between better self-care and a reduced readmission rate [24], self-care behaviour was non-significantly associated with readmission and/or mortality in our study population. This is most probably caused by an on-average high normalized self-care score (median of 68) within the total cohort, with 20% of them being known patients from our outpatient clinic. These patients already received self-care education and were experienced with adjusting their lifestyle, knowledge of their disease and alarming symptoms.

An optimal self-care score always needs improvement [22, 23]. Therefore, it remains important to invest in improving self-care behaviour and to optimize patient education in HF and self-care activities by nurses, during the discharge and outpatient phases.

### Limitations

Several limitations of our analyses should be acknowledged.

A first limitation of this study is the number of participants. However, the sample size turned out to be large enough to demonstrate the prognostic value of the ELAN-HF model. This does however yield a limitation of possible sub-analyses. A second limitation is that using self-reports as in the EHFScBS-9 may be affected by memory and social desirability biases.

## Conclusion

Patients admitted with acute HF have a high risk of post-discharge readmission and death. In this study, we validated the ELAN-HF model that can be used to triage these patients into different risk groups. Based on this knowledge, follow-up treatment in the nurse-led HF clinic can be adjusted in order to improve prognosis.

Self-care behaviour was non-significantly associated with readmission and/or mortality in our study population, most probably due to the fact that most patients were scored as optimal. However, in our opinion, to achieve optimal outcomes, combining risk stratification and applying self-care behaviour is of great importance on the nurse-led HF clinic.

## Data Availability

The datasets used and analyzed during this study are available from the corresponding author on reasonable request.

## Abbreviations

HF: Heart failure
NT-proBNP: N-Terminal pro–B-type natriuretic peptide
SBP: Systolic blood pressure
NYHA: New York Heart Association classification
LVEF: Left Ventricular Ejection Fraction
ELAN-HF: European collaboration on Acute decompensated Heart Failure
EHFScBS-9: European Heart Failure Self-care Behaviour Scale
